# Effectiveness and Community Acceptance of Extending Seasonal Malaria Chemoprevention to Children 5 to 14 Years of Age in Dangassa, Mali

**DOI:** 10.4269/ajtmh.21-0046

**Published:** 2021-11-15

**Authors:** Drissa Konaté, Sory Ibrahim Diawara, Bourama Keita, Nafomon Sogoba, Mahamadou Fayiçal, Agnès Guindo, Sibe Thiam, Sékou Fantamady Traoré, Jeffrey G. Shaffer, Seydou Doumbia, Mahamadou Diakité

**Affiliations:** ^1^ West African International Center for Excellence in Malaria Research, University of Sciences, Techniques and Technologies of Bamako, Bamako, Mali;; ^2^ University Clinical Research Center, University of Sciences, Techniques and Technologies of Bamako, Bamako, Mali;; ^3^ School of Public Health and Tropical Medicine, Tulane University, New Orleans, Louisiana

## Abstract

Seasonal malaria chemoprevention (SMC) was adopted in Mali in 2012 for preventing malaria in children younger than 5 years. Although this strategy has been highly effective in reducing childhood malaria, an uptick in malaria occurrence has occurred in children 5 to 15 years of age. This study aimed to investigate the feasibility of providing SMC to older children. A cohort of 350 children age 5 to 14 years were monitored during the 2019 transmission season in Dangassa, Mali. The intervention group received five monthly rounds of sulfadoxine–pyrimethamine plus amodiaquine, whereas the control group consisted of untreated children. Community acceptance for extending SMC was assessed during the final round. Logistic regression models were applied to compare the risk of *Plasmodium falciparum* malaria infection, anemia, and fever between the intervention and control groups. Kaplan-Meier survival analyses were used to compare the time to *P. falciparum* parasitemia infection between the groups. The community acceptance rate was 96.5% (139 of 144). Significant declines were observed in the prevalence of *P. falciparum* parasitemia (adjusted odds ratio, 0.22; 95% CI, 0.11–0.42) and anemia (adjusted odds ratio, 0.15; 95% CI, 0.07–0.28) in the intervention group compared with the control group. The cumulative incidence of *P. falciparum* infections was significantly greater (75.4%, 104 of 138) in the control group compared with the intervention group (40.7%, 61 of 143, *P* = 0.001). This study reveals that expanding SMC to older children is likely feasible, has high community acceptance, and is in reducing uncomplicated malaria and anemia in older children.

## INTRODUCTION

Despite encouraging reports on the reduction of the burden of malaria, the disease remains a major public health problem in many tropical African countries. According to the 2018 Mali Demographic Health Survey, the prevalence of malaria parasitemia in Mali was 19% in children younger than 5 years old.^1^ Mali is among a group of African countries where the prevalence of malaria remains high.^2^ The WHO recommends seasonal malaria chemoprevention (SMC) administered as sulfadoxine–pyrimethamine (SP) plus amodiaquine (AQ) at monthly intervals during the malaria transmission season in the African Sahel countries to prevent malaria occurrence in children between 3 months and 5 years of age.^3^ In 2012, the Malian National Malaria Control Program (NMCP) adopted this global malaria control initiative formally as local policy.^4^ SMC has been shown to reduce both uncomplicated and severe malaria, and reportedly contributes significantly to the global malaria elimination effort.^5^^–^^7^ Efforts for scaling up SMC are supported in many African countries through the Sahel partnership project.^8^ However, the effectiveness of malaria control interventions in the target population depends on the length of the transmission season and the level of endemicity, particularly for SMC.^9^

Recent studies have shown that successful malaria control in children younger than 5 years of age may have increased the malaria burden in children who are at least 5 years old.^10^^–^^12^ This phenomenon is perhaps because children who are at least 5 years old have received less attention from the NMCP, with malaria control interventions focused primarily on children younger than 5 years of age and pregnant women. The increasing risk of clinical malaria in children older than 5 years has drawn particular attention^9^^,^^13^^–^^15^ particularly in stable and long-transmission areas of sub-Saharan African countries where the control interventions as SMC are designed to target young children.^16^ The aim of this study, therefore, was to assess the efficacy and acceptability of SMC in children as old as 14 years in Dangassa, Mali.

## MATERIALS AND METHODS

### Study site.

This study was carried out in the village of Dangassa, a rural area situated in the Koulikoro region of Mali approximately 82 km southwest of Bamako and 4 km from the Niger River. Malaria transmission occurs year-round in Dangassa, but peak transmission typically occurs between July and December.^17^ Since 2010, Dangassa has served as the primary field study site for the West Africa International Center for Excellence in Malaria Research (ICEMR), which is conducting a large research project with the aim of evaluating the impact of current malaria control interventions on malaria epidemiology. Since 2015, the NMCP, in collaboration with the ICEMR, have implemented the SMC program routinely in Dangassa.

### Study design and population.

In 2017, in collaboration with West Africa ICEMR researchers and local surveillance and clinical staff, a dynamic cohort of 1,400 subjects was assembled from the general population to identify epidemiological factors associated with malaria. An open-label, non-randomized study was conducted to estimate the effect of administering five SMC rounds (as opposed to a current standard of four rounds), implemented in children age 5 to 14 years in Dangassa. The intervention group for this study was selected from a cohort within the ICEMR study, and a control group was selected from the general population. SMC provisions were administered through fixed delivery by medical staff at community health centers as part of routine SMC delivery. Photo identification cards were used to authenticate participant enrollment throughout the study.

### A priori sample size estimates.

A priori sample size estimates were calculated based on the national malaria prevalence among children in rural areas (23%).^1^ Assuming a type I error threshold of 5%, 80% statistical power, a 50% reduction in malaria infection in the intervention group, and a 10% non-response rate, 176 children were needed for each comparison group. For the community acceptability cross-sectional survey, a previous coverage rate of 84% was assumed based on pilot data.^18^ One hundred children in the intervention group were selected randomly every month for the household survey, which yielded 80% statistical power and a 5% type I error rate. During the final round of SMC, clinical questionnaires were used to collect data on community acceptance.

### SMC implementation.

Children younger than 5 years old are eligible to receive SMC free of charge throughout Mali as a standard of care. This study provided SMC for children as old as 14 years using SP and AQ over five monthly rounds in the intervention group between July and November 2019. All children residing in Dangassa during the 2019 transmission season were eligible for the study. Exclusion criteria include known allergic reaction to SP or AQ, chronic illness, or clinical malaria confirmed by rapid diagnostic test during the course of treatment. A therapeutic dose of AQ (10 mg/kg body weight/d), combined with one dose of SP (25 mg sulfadoxine and 1.25 mg pyrimethamine/kg body weight on day 1), was delivered by trained community health workers, who administered the initial dose. The remaining two doses of AQ were administered at home by the parents of the enrolled children.

In July, prior to the first administration of SP and AQ (baseline), and in December (end of study), data were collected on parasitemia, hemoglobin level, body temperature, and use of insecticide-treated bed nets (ITNs) for both the intervention and control groups. During the SMC campaign, routine monitoring and surveillance was performed for all study variables for both comparison groups. Study participants presenting with fever and those who tested positive using rapid diagnostic tests were treated according to standard NMCP guidelines. Household surveys were carried out between 4 and 7 days after each round of SMC to collect and monitor data on adverse events after SMC administration. Community acceptance was assessed via clinical surveys performed during the final round of SMC. Parents of enrolled children were strongly encouraged to bring children experiencing adverse events to a local health center. Adverse events were monitored and tabulated after each of the five rounds of SMC delivery, and parasitemia in blood smears was determined using microscopy. Thick blood films were stained with 10% Giemsa and examined using ×100 magnification and oil immersion of the objective lens of a light microscope. The number of asexual and sexual parasites were counted per 200 leukocytes and per 1,000 leukocytes, respectively. Hemoglobin levels were measured from whole blood using HemoCue Hb 301 analyzers (HemoCue AB, Ängelholm, Sweden). Axillary temperature was measured using electronic thermometers.

### Data collection.

Data responses were captured using electronic case report forms and computer tablets. Daily quality control was performed to detect missing and incorrectly logged data prior to the transfer to a data management center in Bamako, Mali. Range and logic checks were used to ensure all data responses lay within clinically plausible ranges. Data were exported to the STATA statistical package (v. 14.2; STATA Corp., College Station, TX) for data management and statistical analyses.

### Operational definitions.

Prevalence of parasitemia was expressed as the proportion of asymptomatic participants with positive parasitemia confirmed by microscopy. Anemia was defined as hemoglobin < 11 g/dL, and fever was defined as a body temperature of more than 37.5°C. Adverse events were considered to be any reported clinical symptom or complaint after treatment administration. SMC coverage rates were defined as the proportion of children receiving the initial dose of SP and AQ during each round of the SMC campaign.

Interviews with parents of enrolled children were performed using clinical questionnaires to assess SMC acceptability in children at least 5 years of age. These questionnaires included questions regarding the willingness of the parent or guardian to adhere to the SMC strategy and compliance despite its potential adverse events. SMC was deemed acceptable if the parent or guardian reported that the strategy was “good” and generally agreed with the notion of extending the prevention strategy to older children. SMC was deemed unacceptable if the parent reported that the strategy was “bad” and generally did not agree with the notion for extending the SMC program to older children. The clinical questionnaire is provided in Supplemental Appendix SI.

### Statistical analysis.

Data were expressed as proportions and percentages. Logistic regression models were used to estimate the risk of *Plasmodium falciparum* parasitemia, anemia, and fever at baseline (July) and at the end of the study (December) for both comparison groups. Model predictors for the logistic regression models were age group (5–9 years, 10–14 years), use of ITNs (yes or no), *P. falciparum* parasitemia (positive or negative), gametocyte carriage (positive or negative), anemia (yes or no), and fever (yes or no). Cox regression models and Kaplan-Meier curves were used to compare the time to illness onset for *P. falciparum* parasitemia between the comparison groups in children with negative blood smears at baseline. The type I error threshold was set at 5%.

### Ethics approval and informed consent.

This study was carried out through the ICEMR program, in collaboration with the Malian NMCP. The study protocol was approved by the Ethical Committee of the Faculty of Medicine, Pharmacy and Odontostomatology (FMPOS) of the University of Sciences, Techniques and Technologies of Bamako (No. 2019/04/FMPOS). Written informed consent was obtained from all study participants. All malaria cases were treated free of charge at local community health centers by trained personnel as part of routine care.

## RESULTS

### Coverage rates during the SMC campaign.

**Figure 1.**
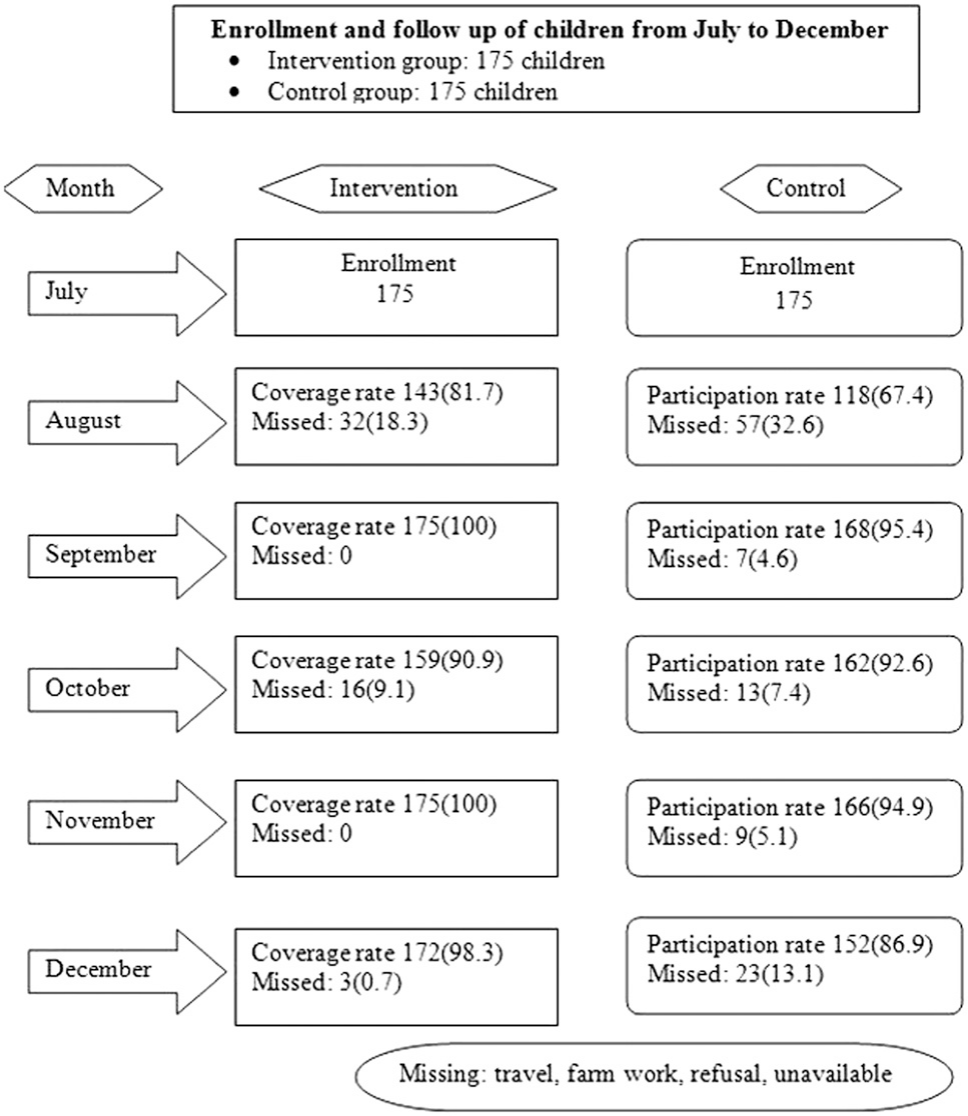
Monthly follow-up of children during seasonal malaria chemoprevention, Dangassa, Mali.

**Figure 2.**
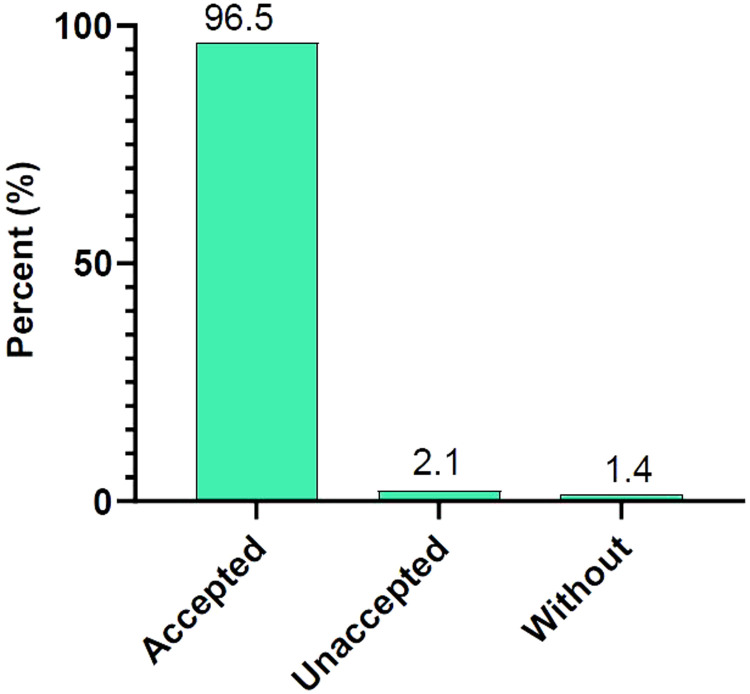
Community acceptance of seasonal malaria chemoprevention, Dangassa, Mali. This figure appears in color at www.ajtmh.org.

Treatment compliance rates were 81.7% (143 of 175) in August, 100% (175 of 175) in September, 90.9% (159 of 175) in October, and 100% (175 of 175) in November. Traveling and farming were reported as the main reasons for missed health facility visits (Figure 1). Among parents or guardians of enrolled children, 96.5% (139 of 144) were amenable to extending SMC to children not currently eligible versus 2.1% (3 of 144) who were not amenable (Figure 2). The most common adverse events reported were vomiting, 11.4% (52 of 457); fever, 11.2% (51 of 457); and weakness, 8.5% (39 of 457; Table 1).

**Table 1:** Reported adverse events following seasonal malaria chemoprevention drug administration

Round	Side effect				
	Fever, *n* (%)	Diarrhea, *n *(%)	Vomiting, *n *(%)	Weakness, *n *(%)	Abdominal pain, *n *(%)
July	12 (17.6)	2 (2.9)	13 (19.1)	6 (8.8)	2 (2.9)
August	4 (5.6)	7 (9.7)	10 (13.9)	4 (5.6)	5 (6.9)
September	8 (11.6)	2 (2.9)	11 (15.9)	3 (4.3)	4 (5.8)
October	9 (9.4)	3 (3.1)	9 (9.4)	4 (4.2)	9 (9.4)
November	18 (11.8)	1 (0.7)	9 (5.9)	22 (14.5)	1 (0.7)
Total	51 (11.2)	15 (3.3)	52 (11.4)	39 (8.5)	21 (4.6)

### Comparison of risk factors between the intervention and control groups.

At baseline (July), no significant differences were observed between the intervention and control groups with respect to *P. falciparum* parasitemia (adjusted odds ratio [aOR], 0.92; 95% CI, 0.53–1.60), fever (aOR, 1.50; 95% CI, 0.18–12.49), and gametocyte carriage (0.0% versus 1.7%, *P* = 0.082). Anemia prevalence was lower in the intervention group than the control group (aOR, 0.55; 95% CI, 0.33–0.91). At the end of the study (December), there was a 78.0% reduction in the prevalence of *P. falciparum* parasitemia in the intervention group (aOR, 0.22; 95% CI, 0.11–0.42). A reduction of 86.0% in the likelihood of having anemia was also observed in the intervention group (aOR, 0.14; 95% CI, 0.07–0.28). Presence of fever (aOR, 0.62; 95% CI, 0.25–1.60), gametocyte carriage (aOR, 1.06; 95% CI, 0.32–3.55), and ITN use (aOR, 1.21; 95% CI, 0.71–2.06) were similar between the two groups (Table 2).

**Table 2:** Logistic regression analyses estimating the prevalence of parasitemia, gametocyte carriage, anemia, and fever by comparison group

Parameter	Baseline (July 2019)					End of study (December 2019)				
	Intervention, *n* (%)	Control, *n* (%)	OR [95% CI]	AOR [95% CI]	*P* value	Intervention, *n* (%)	Control, *n* (%)	OR [95% CI]	AOR [95% CI]	*P* value
Age group, y										
5–9	99 (56.6)	117 (66.9)	–	1	–	97 (56.4)	103 (67.8)	–	1	–
10–14	76 (43.4)	58 (33.1)	1.54 [1.00–2.39]	1.70 [1.08–2.69]	0.02	75 (43.6)	49 (32.2)	1.63 [1.03–2.56]	1.44 [0.81–2.57]	0.2
Use of ITNs										
No	31 (17.7)	53 (30.3)	–	1	–	34 (19.8)	35 (23)	–	1	–
Yes	144 (82.3)	122 (69.7)	2.01 [1.21–3.34]	2.37 [1.40–4.01]	0.01	138 (80.2)	117 (77)	1.21 [0.71–2.06]	1.52 [0.79–2.54]	0.2
Prevalence of parasitemia										
No	143 (81.7)	138 (78.9)	–	1	–	151 (87.8)	97 (63.8)	–	1	–
Yes	32 (18.3)	37 (21.1)	0.83 [0.49–1.41]	0.92 [0.53–1.6]	0.7	21 (12.2)	55 (36.2)	0.25 [0.14–0.43]	0.22 [0.11–0.42]	0.01
Gametocyte carriage										
No	175 (100)	172 (98.3)	–	1	–	166 (96.5)	147 (96.7)	–	1	–
Yes	0	3 (1.7)	–	–	–	6 (3.5)	5 (3.3)	1.06 [0.32–3.55]	1.31 [0.29–5.77]	0.7
Anemia										
No	139 (79.4)	118 (67.4)	–	1	–	154 (89.5)	91 (59.9)	–	1	–
Yes	36 (20.6)	57 (32.6)	0.54 [0.33–0.87]	0.55 [0.33–0.91]	0.02	18 (10.5)	61 (40.1)	0.17 [0.09–0.31]	0.15 [0.07–0.28]	0.01
Fever										
No	173 (98.9)	173 (98.9)	–	1	–	164 (95.3)	141 (92.8)	–	1	–
Yes	2 (1.1)	2 (1.1)	1.00 [0.14–7.18]	1.50 [0.18–12.49]	0.7	8 (4.7)	11 (7.2)	0.62 [0.25–1.60]	1.09 [0.35–3.36]	0.8

AOR = adjusted odds ratio; ITN = insecticide-treated bed nets; OR = odds ratio.

### Prevalence and incidence of parasitemia.

**Figure 3.**
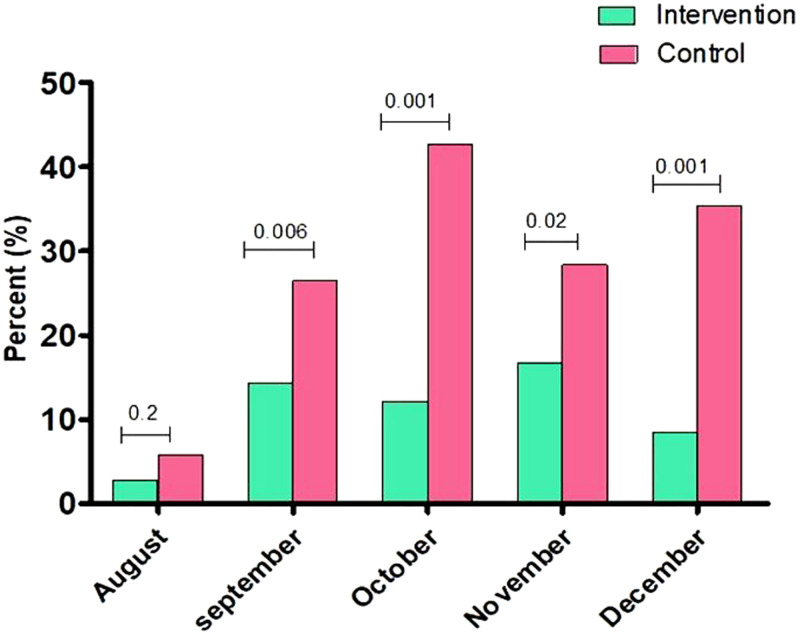
Monthly prevalence of *Plasmodium falciparum* parasitemia. This figure appears in color at www.ajtmh.org.

Declines in the monthly prevalence of *P. falciparum* parasitemia were observed in the intervention group compared with control group in September (14.3% [24 of 168] versus 26.5% [43 of 162], *P* = 0.006); October (12.1% [18 of 149] versus 42.7% [53 of 124], *P* = 0.001); November (16.7% [25 of 150] versus 28.4% [27 of 95], *P* = 0.028); and December (8.5% [12 of 141] versus 35.4% [29 of 82], *P* = 0.001) (Figure 3).

**Figure 4.**
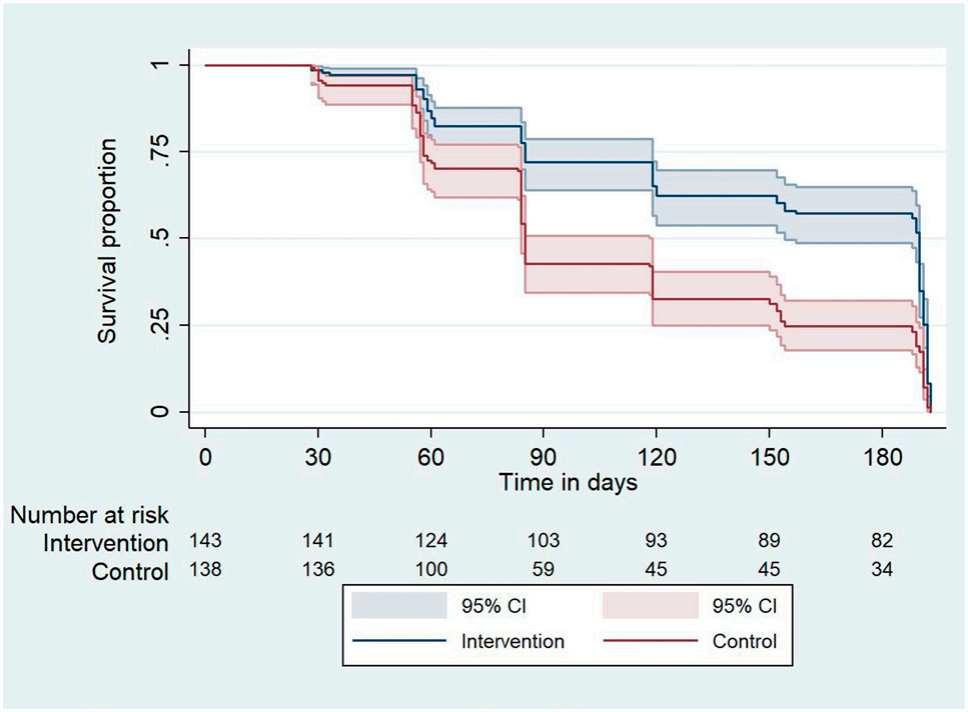
Time to *Plasmodium falciparum* parasitemia infection. This figure appears in color at www.ajtmh.org.

Comparing the first occurrence of *P. falciparum* parasitemia during the transmission season, the cumulative incidence of parasitemia was 60.0% (95% CI, 52–63) in October (during the peak of the malaria transmission season) in the control group versus 28.0% (95% CI, 20–30)in the intervention group. At the end of the study (December), the cumulative incidence was 75.4% (95% CI, 65.0–78.0) in the control group compared with 40.7% (95% CI, 34.0–52.0) in the intervention group (*P* = 0.001, Figure 4). These results suggest that 34.7% of new *P. falciparum* parasitemia cases were averted in the intervention group in children who had negative blood smear at baseline.

## DISCUSSION

In 2012, SMC was adopted in Mali to prevent malaria in children between 3 months and 5 years of age. In collaboration with the NMCP, the ICEMR program began routine deployment of SMC in 2015, which was highly effective in reducing the burden of malaria in children younger than 5 years old.^17^^,^^19^ Because the child malaria burden appeared to increase among older children in some endemic areas,^14^^,^^20^ ICEMR personnel assisted the NMCP to assess the feasibility of extending SMC to children as old as 14 years of age. In addition, this study implemented five monthly SMC rounds. It is worth mentioning that SMC is intended to be used as a preventive therapy, and thus ideally would be implemented prior to the onset of the rainy season and through the end of the transmission season. Dangassa has a long transmission season covering more than 4 months, and therefore a supplementary round was added to cover the peak transmission period. The SMC coverage rates in this study were notably greater than earlier studies in Mali^18^ and Senegal.^13^

The majority of parents of enrolled children favored extending SMC to older children who are not currently covered under the national policy. Positive community acceptance was made apparent to our investigators despite rare occurrences of adverse events. This acceptance is perhaps explained by the strong involvement of the community health workers in SMC delivery, which is known to facilitate treatment adherence and increase coverage rates in the target populations.^13^^,^^21^^,^^22^

At the conclusion of the transmission season, a substantial decrease in the prevalence of malaria parasitemia and anemia was observed in the intervention group, and this finding was confirmed after adjusting for patient age and prior ITN use. Given that clinically meaningful and important reductions in the malaria burden were achieved for children younger than 5 years receiving up to four doses of SP and AQ,^11^^,^^17^^,^^18^^,^^23^ one might expect a greater impact of SMC on reducing malaria occurrence in older children. The rationale for this logic is that the administration of SMC for older children is more straightforward because of easier accessibility during SMC delivery. Older children typically have more opportunities to take their medication without parental assistance, as opposed to younger children whose parental presence is necessary when taking medication.

Our findings reveal that extending SMC delivery to older children was well-tolerated, well-accepted, and effective in reducing the prevalence of parasitemia. These findings are consistent with previous studies reporting SMC effectiveness in children at least 5 years of age.^6^^,^^9^^,^^13^ Straightforward administration of SP and AQ, and high coverage rates of SMC delivery are essential for maximizing their impact on preventing malaria in target populations. In younger children, the administration of drugs requires a lot of parental involvement and is not as straightforward as with older children, which likely leads to under-dosing and thereby decreases the drug effects on parasite growth.

Among children in the intervention group at baseline, a decrease in the monthly prevalence of parasitemia was observed from September to December. Effective reduction in the prevalence of parasitemia and anemia was reported in children younger than 10 years after 5 months of SMC delivery in malaria-endemic areas, which suggests that providing SMC for 5 months during the transmission season is more effective in reducing malaria occurrence in areas with longer transmission seasons.^6^^,^^24^^,^^25^ Comparing the cumulative incidence of *P. falciparum* parasitemia from July to December (period of intense transmission), SMC likely averted 34.70% of new *P. falciparum* parasitemia infections in children who received SP and AQ. The actual effect of SMC on malaria illness and *P. falciparum* parasitemia is associated with the number of SMC rounds received during the campaign and the doses of SP and AQ needed during each round to obtain an optimal plasma concentration to inhibit parasite growth or avoid a new case of *P. falciparum* parasitemia.^19^ Therefore, the provision of SMC to children over five rounds would likely be more effective in reducing the burden of malaria in the target population, particularly in endemic areas with extended seasonal transmission.^13^^,^^26^

The limitations of our study include the non-randomization of study participants in the two groups because the intervention group was selected from the ICEMR study cohort and the control group from the general population. The intervention group was monitored at regular time intervals and thus may have been generally healthier than the general population. However, children in the general population (and children in the control group) had similar access to health care and malaria treatment as a result of the national standard of care. Clinical data were not collected on malaria illness cases and malaria mortality, which could provide additional evidence of the effect of SMC intervention in reducing malaria infection outcomes, including severe malaria.

It is worth mentioning that extending SMC to older children would increase the cost of the SMC programs. Therefore, it is important to reproduce the results from this study on a larger scale and to evaluate its cost-effectiveness before the findings are used to guide public policy.

## Supplemental Material


Supplemental materials
