# Supplementary material for: Effectiveness and Community Acceptance of Extending Seasonal Malaria Chemoprevention to Children 5 to 14 Years of Age in Dangassa, Mali
Source: Am J Trop Med Hyg. 2021 Nov 15;106(2):648–54. doi: 10.4269/ajtmh.21-0046 (PMC8832934; doi:10.4269/ajtmh.21-0046)
Supplement: Supplementary file 1 [file tpmd210046.SD1.pdf]

STUDY STUDYID		SUBJECTID USUBJID		SITE SITEID	Dangassa

SMC adherence and compliance

VISIT DATE[1] [2]: |__|__|-|__|__|__|-|__|__|__|__| (DD-MMM-YYYY) VISDAT	VISIT 1 ☐ 2 ☐ 3☐ 4☐ 5☐
Personne interrogée : /_____/	1=Père 2=Mère 3=Tuteur 4=Autre (préciser) :_________
Age groups	1☐ (3-59 month) 2☐(60-119 month)

Adhésion au traitement

Dans les 7jours précédents l'enfant a-t-il été vu par l'agent de santé pour la campagne de la CPS ?	|____| 1=Oui 2=Non
Si non, quelle en était la principale raison ?	|____| 1=Voyage 2=Trop occupé/non disponible pour amener l'enfant 3=Oubli du jour de la campagne 4=Ne savait pas où aller 5=L'agent de santé n'a jamais visité la maison 6=Enfant malade 7=Autre ____________
L'enfant a-t-il reçu une carte CPS ?	Oui ☐ Non ☐
Est-ce que la carte CPS de l'enfant est disponible ?	Oui ☐ Non ☐
Si non, donner les raisons ?	|____| 1=Perdu 2=Parent/tuteur absent 3=Carte non remise 4=Autres :__________
Vérifiez sur la carte CPS si l'enfant a reçu SP+AQ pour ce passage	|____| 1=Reçu, 2=Non reçu
Si carte non vue, l'enfant a-t-il quand même reçu la dose SP+AQ selon les parents ?	☐ Reçu ☐ Non reçu
Si SP+AQ non reçu sur la carte, pourquoi ? *Donner la principale raison.*	|____| 1= Enfant malade 2=Allergie au médicament 3= L'agent n'a pas noté sur la carte 4=Autre ___________________
La dose de J1 a-t-elle été vomie /rejetée ?	Oui ☐ Non ☐
Si la dose a été vomie /rejetée une dose de remplacement a-t-elle été donnée ?	Oui ☐ Non ☐
Si une dose de remplacement du 1er jour a été donnée, cette dose a-t-elle été vomie/rejetée ?	Oui ☐ Non ☐
Est-ce que l'enfant a pris la dose de médicament du 2ème jour au cours de ce passage ?	Oui ☐ Non ☐
Si médicament du 2ème jour n'a pas été pris, pourquoi ?	|___| 1=Oubli de donner 2=Dose non retrouvée

[1] Visit date pertains to all information collected on day of enrolment / visit 1
[2] All dates to be recorded as DD-MMM-YYYY

STUDY<br>STUDYID		SUBJECTID<br>USUBJID		SITE<br>SITEID	Dangassa

Donner la principale raison.	3=Dose donnée à un autre enfant<br>4=Veut garder pour une maladie future<br>5=Autre___________________
La dose du 2ème jour a-t-elle été vomie/rejetée ?	Oui ☐ Non ☐
Une dose de remplacement du 2ème jour a été donnée ?	Oui ☐ Non ☐
Est-ce l'enfant a reçu la dose du 3ème jour des médicaments de la CPS au cours de ce passage ?	Oui ☐ Non ☐
Si le médicament du 3ème jour n'a pas été pris, pourquoi ?	|____| 1=Oubli de donner<br>2=Dose non retrouvée<br>3=Dose donnée à un autre enfant<br>4=Veut garder pour une maladie future<br>5=Autre___________________
La dose du 3ème jour a-t-elle été vomie/rejetée ?	Oui ☐ Non ☐
Si une dose de remplacement du 3ème jour a été donnée, cette dose a-t-elle été vomie/rejetée ?	Oui ☐ Non ☐
Est-ce que je peux voir le reste des médicaments que vous deviez donner à l'enfant ?	|___| 1=Reste 2 doses<br>2=Reste 1 dose<br>3=Emballage vu et vide<br>4=Ni dose ni emballage disponible
**Effets secondaires et conduite à tenir**	
Est-ce que l'enfant a fait de la fièvre dans les 2 derniers jours avant de voir l'agent de santé au cours de la campagne de la CPS ?	Oui ☐ Non ☐
Si fièvre, est-ce que l'agent de santé a pris du sang de l'enfant au doigt ou au talon à l'aide	Oui ☐ Non ☐
Quel a été le résultat du test ?	☐ positif ☐ négatif ☐ ne sait pas
Est-ce que l'agent de santé a donné des médicaments pour la maladie de l'enfant ?	Oui ☐ Non ☐
Quels médicaments l'agent de santé a donné à l'enfant<br><br>*Montrer des échantillons de ces médicaments*	|____| 1=SP-AQ (Médicament de la CPS)<br>2=AL (Artéméther – Luméfantrine)<br>3=Quinine<br>4=Autre ______________________
Est-ce que l'enfant a eu les symptômes suivants depuis le 1er jour d'administration des médicaments de la CPS?<br><br>*Cocher dans toutes les cases correspondant aux symptômes cités*	1=Diarrhées /____/<br>2=Vomissements /____/<br>3=Fatigue/ faiblesse /____/<br>4=Douleur abdominale /____/<br>5=Démangeaison /____/<br>6= Ictère/jaunisse /____/<br>7=Autre ______________<br>8=Aucun /____/

STUDY<br>STUDYID		SUBJECTID<br>USUBJID		SITE<br>SITEID	Dangassa

Pensez-vous que ces symptômes étaient liés à la prise des médicaments de la CPS ?	Oui ☐ Non ☐
Avez-vous fait consulter l'enfant quelque part pour ces symptômes ?	Oui ☐ Non ☐
Si oui, combien de temps avez-vous pris pour aller consulter ?	/_____/<br>1=le même jour<br>2=le jour suivant<br>3=plus de deux jours
Si non, pourquoi ?	Ca va passer /____/<br>Manque de moyen /____/<br>Autres ________________________<br>Rien /____/
L'enfant a-t-il dormi sous une moustiquaire imprégnée d'insecticide la nuit dernière?	Oui ☐ Non ☐

**Opinion sur la CPS**	
Pensez-vous que la CPS est :	☐ Très bonne ☐ Bonne ☐ Mauvaise ☐ Sans opinion
Pensez-vous que la CPS devrait continuer dans le futur ?	☐ Oui ☐ Non ☐Ne sait pas
Pensez-vous qu'il y a des choses à améliorer pour que la CPS soit encore plus efficace dans le futur ?	____<br>____
Pensez-vous que les agents distributeurs font correctement leur travail ?	____<br>____
Avez-vous des commentaires ?	
